# Describing associations between child maltreatment frequency and the frequency and timing of subsequent delinquent or criminal behaviors across development: variation by sex, sexual orientation, and race

**DOI:** 10.1186/s12889-019-7655-7

**Published:** 2019-11-12

**Authors:** Hannah Lantos, Andra Wilkinson, Hannah Winslow, Tyler McDaniel

**Affiliations:** 1 Child Trends, 7315 Wisconsin Ave, Suite 1200W, Bethesda, MD 20814 USA; 2 Department of Population, Family, and Reproductive Health, Johns Hopkins Bloomberg School of Public Health, 615 N. Wolfe St, Baltimore, MD 21205 USA; 3 Department of Maternal and Child Health, University of North Carolina Gillings School of Global Public Health, 135 Dauer Drive, Chapel Hill, NC 27599 USA; 4 Sociology Department, Graduate Student, Stanford University, 450 Serra Mall, Building 120, Room 160, Stanford, CA 94305-2047 USA

**Keywords:** Maltreatment, Abuse, Delinquency, Violent, Nonviolent

## Abstract

**Background:**

Child maltreatment has been linked to lower health, education, and income later in life, and is associated with increased engagement in delinquent or criminal behaviors. This paper explores trajectories of these behaviors from adolescence into early adulthood and tests maltreatment as a predictor, and whether observed patterns are consistent across different demographic groups.

**Methods:**

Using data from the National Longitudinal Study of Adolescent to Adult Health, a longitudinal study of a nationally representative sample of U.S. adolescents (in grades 7–12 in the 1994–95 school year), we ran linear mixed effects models to estimate growth curves of two dependent variables: violent and nonviolent offending behavior. We tested if maltreatment altered the intercept or slope of the curves and how the curves of these behaviors and the associations between them and maltreatment varied by sex, race/ethnicity, and sexual orientation.

**Results:**

The sample (*n* = 10,613) had equal proportions males and females, approximately one third identified as a race/ethnicity other than white, and over 10% were non-heterosexual. Experiences of maltreatment were highest for Native Americans and lowest for whites. Models indicated that males were more likely than females to engage in both violent and nonviolent offending and respondents who identified as non-heterosexual were more likely than their heterosexual peers to engage in nonviolent offending behavior. When maltreatment was included in models as a predictor, adolescents who experienced maltreatment had a more rapid increase in their non-violent offending behavior. For violent offending behavior, adolescents who experienced maltreatment had higher levels of offending and the levels progressively increased as maltreatment frequency did. Sex was a moderator; the relationship between maltreatment and predicted nonviolent offending was stronger for males than it was for females. Race/ethnicity and sexual orientation did not moderate the associations between maltreatment and offending behavior.

**Conclusions:**

This study provides insights from a nationally representative sample into the pattern of both delinquent and criminal behaviors in adolescence and young adulthood, describing not only how the pattern varies over time, but also by sociodemographics and offending type. Additionally, it highlights how the association between maltreatment and these behaviors varies by both offending type and sex.

## Background

The most recent data on child maltreatment in the United States indicates that 9.1 out of 1000 children have experienced maltreatment that resulted in their involvement in the child welfare system [1]. Federal data collection efforts in the United States track multiple types of maltreatment over time – defined as abuse (including physical, sexual, emotional, or psychological), exploitation, or neglect perpetrated by someone who has power over a child (e.g., a parent, teacher, clergy member, or other caregiver) [2]. Neglect is by far the most common type of maltreatment experienced among those children tracked by the federal government. Just over three-quarters of children who have been maltreated have experienced neglect while nearly one in five (18%) cases report physical abuse and nearly one in ten (9%) report sexual abuse. Other types of maltreatment such as emotional abuse, a parent’s substance abuse, or a lack of supervision were experienced by nearly 11% of the children. As the percentages show (by adding up to more than 100), many children experience multiple types of maltreatment (14%), with the most common combination being physical abuse and neglect (5%) [3].

While the immediate effects of maltreatment are clear, there are many ways that maltreatment continues to affect children’s well-being. Maltreatment and experiences of violence impact children’s well-being long into the future – affecting their physical and emotional health [4–6], school attainment [7], and socioeconomic stability [8] into adulthood. Additionally, there is risk of a link between experiences of maltreatment and engagement in delinquent behaviors during childhood and adolescence [9]. Children who are exposed to maltreatment are more likely to engage in delinquent behavior later, such as stealing or committing violence [10]. A study of a nationally representative group of 14-year-olds found that an adolescent may be 40–60% more likely to engage in delinquent behavior if they were maltreated as a child [11]. Additionally, among justice-involved youth – youth who have been caught and convicted of criminal delinquency – 40-90% of girls and 25–65% of boys are estimated to have experienced maltreatment in childhood [12]. Note that we use the terms “delinquent or criminal behavior” as well as “offending behavior” or “offenses” in this paper. This is because delinquent behavior refers to youth under the age of 18 while for adults, these behaviors are often criminal offenses. Thus, these terms are used throughout the paper to capture the changes in behavior across ages.

Notably, there are two questions embedded in these statistics that are different in important ways: 1) of the kids who experienced maltreatment, how many (and who) will go on to engage in delinquent or criminal behavior versus 2) of those who have engaged in delinquent or criminal behavior, how many experienced maltreatment? There is a long record of scholarship focused on these questions with papers using data from both larger studies as well as from small, homogenous, high-risk samples. For example, one study found relationships between maltreatment and violent delinquency and studied variation in these associations driven by different types of maltreatment [13] while a second one looked at the developmental processes underlying “aging out” of crime [14]. Another one looked specifically at the immediate and long-term associations between violence exposure and delinquent behavior finding long-term associations that are attenuated over time [15].

In this paper, we focus on the first question because our data enable us to explore these questions in the general population. Using this large, nationally representative sample, we are able to stratify by multiple races as well as by sexuality – sub-samples which are often too small in other samples. We also are able to study the specific timing of the delinquent behavior across a young person’s life by modeling growth curves across ages. This is possible in our study because respondents were not all the same age in Wave I, meaning we are able to include respondents of all ages from ages 12 to 30.

The present study uses longitudinal data from a large nationally representative study of adolescents who were followed into young adulthood. As the data source included not only multiple types of maltreatment and delinquent and criminal behaviors, but also their frequency, the present study was able to use linear mixed effects models to examine the relationship between maltreatment and these behaviors across ages. The two research questions and hypotheses we proposed were as follows:

1. What is the relationship between childhood maltreatment and delinquent or criminal behaviors from adolescence into young adulthood?

Hypothesis: Increased frequency of maltreatment experiences will be associated with a higher frequency of non-violent and violent offending frequency across development than the pattern observed for youth who did not experience childhood maltreatment [16–20].

2. Does this relationship vary by sex, race/ethnicity, and sexual orientation and if so, how?

Hypothesis: The positive association between maltreatment frequency and delinquency frequency to be moderated by sex, race/ethnicity, and sexual orientation such that the association would be stronger for females, youth of color, and LGBTQI youth compared to their male, white, or straight peers [16, 21–23].

Our use of the robust method of linear mixed effects models allowed exploration of these trajectories and how they differ by race, sex, and sexual orientation. Additionally, while using self-reported versus administrative data on experiences of maltreatment both have their strengths and weaknesses [24], this paper uses self-reported data for both reports of maltreatment and delinquent behavior. The data come from a large, well-known, nationally representative, longitudinal sample and allow us to explore these associations.

## Methods

### Sample

The present study used data from the National Longitudinal Study of Adolescent to Adult Health (Add Health), a longitudinal study of a nationally representative sample of U.S. adolescents who were in grades 7–12 in the 1994–95 school year (Wave I, adolescence). There have been four in-home interviews to date. The sample used in these analyses was restricted to respondents interviewed at Waves I, III (ages 18 to 26, emerging adulthood), and IV (ages 24 to 32, young adulthood), with valid sampling weights (*N* = 12,288) and who had complete data on all variables of interest (*N* = 10,613, 86%). Data from Wave II were not used as Wave I high school seniors were not followed by design. Details of the Add Health study and design are described elsewhere [25]. Our secondary analyses were reviewed by the Institutional Review Board at Child Trends and deemed exempt.

### Measures

#### Independent variable: childhood maltreatment frequency

Childhood maltreatment was measured via a categorical variable capturing frequency (0 [never] – 10 [10 or more times]) of experiencing emotional, physical, or sexual abuse before age 18 or physical or supervisory neglect before sixth grade by a parent or an adult caregiver. This variable captures frequency of maltreatment rather than type because recent evidence suggests the chronicity of maltreatment is potentially a better indicator of negative consequences than the type of maltreatment (severity is not measured in Add Health) [26]. The average maltreatment frequency in our analytic sample was 2.6 times with a standard deviation of 2.7.

#### Dependent variable: frequency of delinquent and criminal offenses

Offense frequency was measured at each wave via two scales of frequency in the past 12 months, one for both violent and nonviolent offenses, mirroring prior measures of offenses using Add Health data [27, 28]. Violent offense frequency (alpha = .60–.73, across the waves) included the following indicators at each wave: shooting or stabbing someone; hurting someone badly enough to need bandages or care from a doctor or nurse; using or threatening a weapon to get something from someone; pulling a knife or gun on someone; and being in a group fight. In adolescence (Wave I), the mean frequency of committing violent offenses in the past year was 0.72 (or less than one average violent offense per year), and by young adulthood (Wave IV), the mean frequency dropped to 0.19.

Nonviolent offense frequency (alpha = .50–.66, across the waves) included the following indicators at each wave: deliberately damaging property that didn’t belong to you; going into a house or building to steal something; stealing something worth less than $50; stealing something worth more than $50; selling marijuana or other drugs; and taking an illegal drug using a needle. The choice of indicators was constrained by what items were included in the survey, which were included in each Wave; and if items fit better conceptually as control variables. In adolescence, the mean frequency of nonviolent offenses in the past year was 0.86 and this dropped to 0.25 by young adulthood.

#### Control variables

Previously published relevant analyses were reviewed to inform the type of potential confounders that should be controlled for [29–31]. Sociodemographic variables included sex and race/ethnicity from Wave I (Hispanic and non-Hispanic White, Black, Asian, Native American, and Other), and sexual orientation/attraction (respondent included if they identified as homosexual or bisexual or if they reported attraction to the same sex) at Wave III. Trouble in school was measured with an indicator of whether the respondent had ever repeated or been held back a grade and another indicator if they had ever been suspended, expelled, or dropped out. An indicator of whether anyone in the household had received public assistance before the respondent was 18-years-old was used to approximate the socioeconomic status of their childhood home. Whether the respondent had ever lived in a foster home was also included. Finally, any use of substances before Wave I was controlled for, including alcohol, cigarettes, marijuana, and other illicit substances. Injection drug use was not included in this measure as it was included in the nonviolent offense frequency measure.

### Analyses

The data set was structured by age instead of wave to capture the developmental trajectory from adolescence to young adulthood. Linear mixed effects models were used to estimate growth curves of the two dependent variables: frequency of either violent or nonviolent delinquent or criminal behaviors. These models allowed for estimation of change over time while controlling for unobserved time-invariant characteristics that could confound any associations. Nine models were fit for each of the two dependent variables. The first five models were used to estimate patterns of offenses starting with an unadjusted model, adding covariates, and testing moderation of the base pattern of offenses by sex, race/ethnicity and sexual orientation. The next four models test a temporal association, whether childhood maltreatment is significantly associated with the starting point and trend in the growth curve of offenses, and whether the association varies by sex, race/ethnicity, or sexual orientation.

All significant models were run with a random intercept and slope to examine variation in the effect. The intraclass correlation coefficient (ICC), used in linear mixed effects models to determine the percentage of variance in offense frequency that is due to variance between individuals was used in these analyses. However, the sampling weights for analyzing the Add Health data inhibit testing if the ICC is significantly different than zero. So, the ICC from the first and the last model were compared to determine how much of the variance in offense frequency was explained by the predictor variables.

## Results

The analytic sample (Table 1) was comprised of equal proportions of males and females. Approximately one-third of the sample were young people of color. Over 10% of the sample reported sexual attraction to either both sexes or the same sex and/or reported their sexual orientation as something other than 100% heterosexual. The majority of the sample (77.0%) had experienced at least one type of maltreatment in childhood. Nearly one-third (32.5%) of the sample had committed nonviolent offenses and 30% had committed violent offenses during their adolescence (Wave I).

**Table 1:** Demographic summary of analytic sample, including total Ns and sample percentages by category

	N or mean	Weighted % or SD
Sex		
Male	5373	50.6%
Female	5240	49.4%
Race/ethnicity		
Hispanic	1249	11.8%
Black	1600	15.1%
Asian	375	3.5%
American	217	2.0%
Other	102	1.0%
White	7070	66.6%
Sexual Orientation		
LGBQ	1305	12.3%
Age at Wave I	15.4	1.8
Age at Wave III	21.8	1.9
Age at Wave IV	28.3	1.9
Nonviolent offending (any)		
Nonviolent offending frequency at Wave I	3449	32.5%
Nonviolent offending frequency at Wave III	1983	18.7%
Nonviolent offending frequency at Wave IV	1145	10.8%
Violent offending (any)		
Violent offending frequency at Wave I	3113	29.3%
Violent offending frequency at Wave III	1308	12.3%
Violent offending frequency at Wave IV	650	6.1%
Maltreatment (any)	7145	67.3%
Control Variables		
Public assistance in household before age 18	1673	15.8%
Ever repeated or been held back a grade	2150	20.3%
Ever suspended, expelled or dropped out	142	1.3%
Ever used alcohol, cigarettes, or illicit substances	6181	58.2%
Ever in a foster home	173	1.6%

The key predictor and outcome variables showed variation by sociodemographic characteristics (Table 2). The average childhood maltreatment frequency was highest for Native Americans and lowest for Whites in adolescence (M = 3.56 vs. 2.54, respectively). Average nonviolent delinquency frequency in adolescence was higher for Lesbian, Gay, Bisexual or Queer (LGBQ) youth compared to their non-LGBQ counterparts (M = 1.10 vs. 0.82). For violent delinquency, average frequency in adolescence was again highest for Native Americans and lowest for Whites (M = 1.26 vs. 0.57). Average frequency of both nonviolent and violent delinquency was higher for males compared to females during adolescence.

**Table 2:** Average maltreatment, nonviolent offense and violent offense frequency by demographic descriptors

	Average maltreatment frequency		Average nonviolent offense frequency in adolescence		Average violent offense frequency in adolescence	
	Mean	Std. Dev.	Mean	Std. Dev.	Mean	Std. Dev.
Sex						
Male	2.51	2.48	1.13***	1.93	0.97***	1.87
Female	2.78***	2.92	0.58	1.47	0.45	1.28
Race/ethnicity (white = referent)						
Hispanic	2.88**	3.22	1.07*	2.33	1.13***	2.74
Black	2.64	3.10	0.65*	1.78	1.01***	2.28
Asian	3.31***	4.03	0.97	2.65	0.63	2.06
Native American	3.56***	3.18	1.26*	2.05	1.26***	2.11
Other	2.52	2.12	1.09	2.11	0.58	1.62
White	2.54	2.40	0.84	1.58	0.57	1.24
LGBQ						
No	2.53	2.65	0.82	1.74	0.72	1.67
Yes	3.40***	2.93	1.10***	1.99	0.67	1.59

*p-value< 0.05; **p-value< 0.01; and ***p-value< 0.001 in post hoc testing

**Fig. 1:**
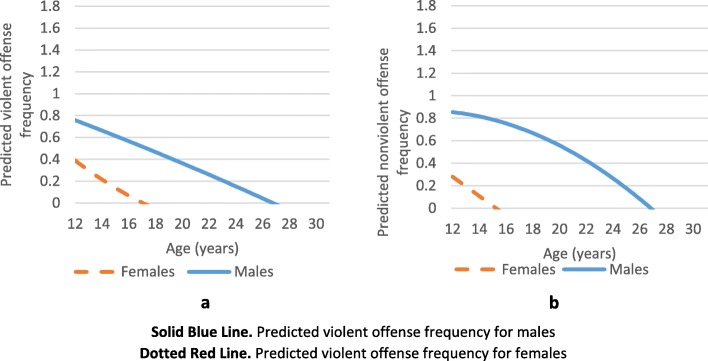
**a** Differences in predicted violent offense frequency by sex. **b** Differences in predicted nonviolent offense frequency by sex

The results of our analyses can be broken down into two main parts: in the first, we studied the pattern of delinquent and criminal behavior by age; in the second, we studied the relationship between maltreatment and these behaviors. In the first part, we found that the trend in the frequency of these behaviors declines steadily from adolescence into young adulthood. When examining variation in offending frequency by sociodemographic variables, there appears to be a significant difference by sex, with males having consistently higher predicted offending frequencies than females across development, for both violent (Fig. 1a) and nonviolent offending (Fig. 1b) (Additional file 1: Table S1 and Additional file 2: Table S2 show the model results in table form and Additional file 3: Table S3 shows the intraclass correlation for both violent and nonviolent offending models comparing baseline and analytic models).

**Fig. 2:**
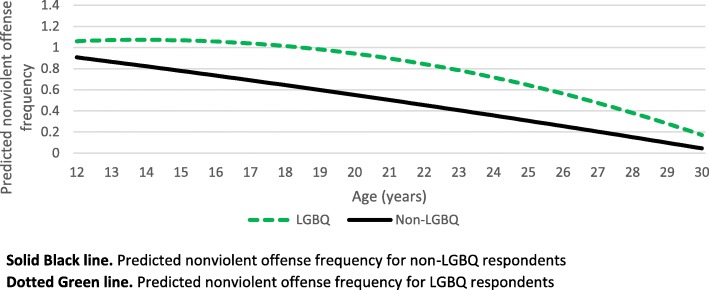
Differences in predicted nonviolent offense frequency by sexual orientation

For other sociodemographic categories, we found evidence of significant moderation by sexual orientation such that individuals identifying as LGBQ had significantly higher predicted nonviolent offending frequency across development compared to non-LGBQ individuals (Fig. 2).

In the remaining tests for moderation by sociodemographics (race/ethnicity), the results, though statistically significant, were not robust enough to be shared. For example, there were only statistically significant differences in the tails of the graphs and thus the images make the differences look more robust than they likely are.

**Fig. 3:**
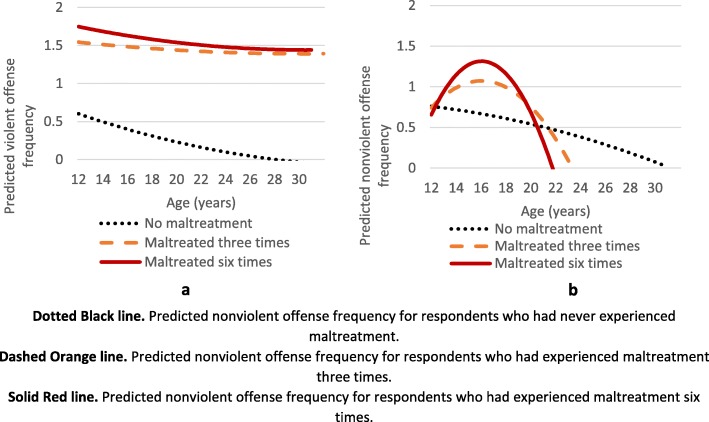
**a** Differences in predicted violent offense frequency by experience of maltreatment. **b** Differences in predicted nonviolent offense frequency by experience of maltreatment

In the second part of our analyses, we examined the association between childhood maltreatment and nonviolent and violent delinquent and criminal offending frequency across age. Specifically, our results focus on whether the level or rate of change differed across maltreatment status. Figures 3a and b below demonstrate that maltreatment significantly alters the pattern of predicted offending frequency across age. For violent offending (Fig. 3a), any maltreatment moderates the relationships such that increased frequency of maltreatment is associated with more delinquent behavior. For example, those who experienced maltreatment start one whole instance of predicted violent delinquency higher in early adolescence; moreover, the gap persists into adulthood and does not appear to vary much by maltreatment frequency. For nonviolent offending (Fig. 3b), maltreatment frequency moderates the rate at which youth engage in delinquent behavior over time. For those who experienced maltreatment, the rate of change (slope) in predicted nonviolent offending frequency increases in early adolescence and peaks in the later teenage years; this increase is steeper and peaks at a higher point as maltreatment frequency goes up (solid, red line in Fig. 3b). Maltreatment frequencies of three and six were chosen for the figures as they were commonly reported frequencies by respondents.

**Fig. 4:**
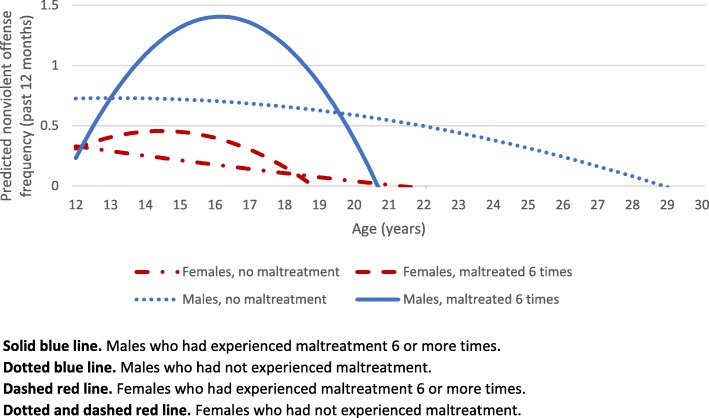
Differences in predicted nonviolent offense frequency (past 12 months) by sex and maltreatment frequency

Breaking down these relationships further, we found significant moderation by sex such that the relationship between maltreatment and predicted nonviolent offending is stronger for males compared to females. In Fig. 4, below, we see the gap between the blue lines (for males) is much larger than the gap between the dashed red lines (for females). The solid blue line peaks with teenage maltreated males having the greatest predicted nonviolent offense frequency. For violent offending, we only found evidence for moderation by sex in the absence of maltreatment.

We found no evidence suggesting that race or sexual orientation moderate the relationship between maltreatment and nonviolent or violent offense frequency. Comparing the ICCs across the respective models, we noted decreases in the ICC, indicating the predictor variables were explaining some of the variance in offending frequency. For example, the ICC baseline model for violent offending frequency (M1) indicates 16% of the variance in violent offending frequency is due to variance between individuals. The predictor variables added in subsequent models reduced this to 10%, meaning the bulk of the variance in violent offending is within individuals over time, rather than between them. The ICC for nonviolent offending models also decreased from 19 to 13%. Random effects by intercept and slope did not add meaningful variation to any of the demonstrated models.

## Discussion

Findings from this research uncovered patterns in the relationship between maltreatment and later delinquent and criminal behaviors from adolescence into young adulthood and how these patterns vary by sociodemographics. Specifically, we explored how maltreatment frequency affected the starting point and trajectory over time in predicted offense frequency from ages 12 to 30 and how this pattern varied by sex, race/ethnicity, and sexual orientation.

To answer our first research question, we found that those youth who had experienced maltreatment were more likely to engage in violent offending behavior, a finding backed up by previous research [32–34]. We also found that youth who experienced maltreatment were more likely to see a faster increase in the amount of non-violent offending they engaged in. While these are two different components that describe increased offending frequency, they align with our hypothesis that increased maltreatment experiences would be associated with both increased violent and non-violent offending behavior.

This paper also extends our understanding of the relationship between maltreatment and offending later in development. More frequently than exploring the relationship between childhood maltreatment and offending behaviors into adulthood (which has been explored minimally), papers explore the linkage between early childhood experiences of abuse, neglect, or trauma with long-term health outcomes or self-sufficiency [35–37]. Prior papers with delinquency outcomes have mostly focused their studies on adolescence or early adulthood (often age 21) [21, 32–34, 38]. The analyses here examined whether the decline in criminal behavior that we see in the administrative data extends through the 20’s following maltreatment. Prior papers also tend to have outcomes at specific ages for the whole dataset (e.g., 15–19), and we build on this by having data from respondents at different ages to show the shape of trends from age 12 to 30 and allow for nonlinearity such that we can see that predicted offending frequency peaks at around age 16.

To answer our second question, we explored differences by sex, race/ethnicity and sexual orientation. We did not find differences by race/ethnicity or sexual orientation. Our findings indicate that the link between maltreatment and later offending varies significantly by sex. Specifically, results showed differences in nonviolent offending between males and females, such that, among those who experienced maltreatment, the predicted nonviolent offense frequency was significantly higher for males compared to females. This was contrary to our hypothesis where we expected that even while males commit more offending behavior that the association with maltreatment would be stronger for females. Recent explorations of a similar question have found that the associations can vary across gender by type of maltreatment [39]. These findings have implications for the dialogue surrounding male-perpetrated offending because given recent research into trauma and externalizing behavior [40, 41], understanding males’ experiences of maltreatment could help motivate the provision of needed therapeutic treatment or positive relationships that could reduce negative behaviors [42, 43]. These findings may also shed light on the notions around gender and risky or offending behavior. The stronger relationship between maltreatment and nonviolent offense frequency for males indicates that the higher rate of offending among boys may not only be due to their higher proclivity for risk behavior but also due to an externalizing response to maltreatment. This finding is consistent with previous literature that demonstrates externalizing responses (e.g., delinquency) are more common for males, compared to the internalizing responses (e.g., depressive symptoms) that are more common for females [44, 45].

One important finding in this paper is that there are no differences seen for the relationship between maltreatment and either violent or nonviolent offending by either race/ethnicity or sexual orientation. Previous research with administrative samples has found a linkage by race [12] while other prospective studies also found no linkages between maltreatment and violent behavior by race [46]. We see this as positive in many ways. For instance, the lack of difference indicates that there is not one particular race or sexual orientation where maltreatment is associated with more subsequent offending, violent or nonviolent. More specifically, all youth – regardless of race/ethnicity or sexual orientation – negatively respond to maltreatment. These findings are not necessarily surprising given that it is likely that humans have universal biological and adaptive responses to maltreatment during childhood including how it affects their brains, emotions, and cognitive processes [47–50]. Rather, they should prompt us to think more broadly about trauma and children’s behavior within the specific context in which they live, allowing us to respond more appropriately to their needs given their specific environmental exposures.

We also hypothesized that LGBQ youth may struggle with their mental health and exhibit more externalizing behaviors [27, 51, 52]. We did not see this in our results. (Note that while we did find small differences in nonviolent offending behavior by sexual orientation that these differences were found overall and were not based on different past experiences of maltreatment. Specifically, youth who identified as heterosexual or homosexual did not report different patterns of offending behavior following experiences of maltreatment than their straight peers. Therefore, while their behavior may be externalizing following other struggles, there do not appear to be differences in externalizing behavior following maltreatment by sexual orientation.) This may indicate either that non-heterosexual youth are doing better overall than we hypothesized and are more similar to their heterosexual peers, or that their struggles are more likely to be exhibited with internalizing symptoms rather than externalizing symptoms [27, 53, 54].

Finally, we hypothesized that we may see differences across race due to different stressors and violence exposure. Despite finding no variation in delinquent or criminal behavior following experiences of maltreatment for adolescents and adults across race, there is substantial evidence for differential treatment after criminal or delinquent behavior occurs. Past studies find that both Black and Latino students are significantly more likely to receive a suspension in comparison to their white counterparts, a discrepancy that appears as early as preschool [55, 56]. This trend continues through adolescence when Black and Latino individuals are more likely to have both contact with police as well as experience arrest and engagement in the juvenile justice system [57, 58]. This is particularly true for boys. Our findings, coupled with past literature, reinforce the need to reexamine areas where inequalities in the trajectory from maltreatment to juvenile delinquency and offending persist so that we can create a more equitable juvenile and adult justice system.

There are several limitations to the analyses. Specifically, while the most recent round of Add Health data is brand new (2016–2018) [59] (we do not use this most recent wave), the respondents are now in their late 30s and early 40s, meaning that the experiences of maltreatment that we are analyzing happened some time ago. Fortunately, reports of childhood abuse and neglect have been declining in the last two decades [60]. This could mean that the relationships we see here may differ in a sample of youth who experienced maltreatment today; however, we also have seen delinquency decrease significantly over the same time period, bolstering the argument that these experiences and behaviors may be intertwined [43].

Additionally, exploring the linkages between specific *types* and frequencies of maltreatment with specific offending behaviors may be an important next step which we did not do here. Watts and Iratzoqui do look at this by gender in their new paper [39], which explored moderation by gender in how different types of abuse or neglect are associated with different types of delinquency. More research along this strain of questioning could shed light on whether certain types of maltreatment have a stronger relationship with certain types of offending and deserve more attention.

In addition to these challenges, the Cronbach’s alphas for the offending frequency measures were as low as 0.5 at one of the waves, which indicate low internal consistency reliability of our outcome measures, particularly for non-violent offending behavior at Wave I. Previous analyses of offending behavior using these data have constructed similar measures, so we used these measures to remain congruent with the broader field [28]. It makes sense the different behaviors measured by the non-violent offending scale would have lower internal consistency reliability than the violent offending scale as the behaviors in the former cover a wide range of behaviors (e.g., trespassing, theft, and injection drug use). By comparison, the behaviors measured in the violent offending scale seem more conceptually congruent as they all involve violent behaviors. Finally, while we mentioned above that there are pros and cons to self-report data, some research indicates that self-reported retrospective data is more likely to overestimate associations with self-reported outcomes. As our outcomes are self-reported, this is something to consider [61].

There are also strengths to these analyses. First, we are also to stratify by race/ethnicity and sexual orientation because of the sample size, and our data cover nearly 20 years of age. Second, the lack of variation from random effects in the intercept and slope indicate the sample results are well represented by the predicted plots. In other words, if we allowed the predicted lines to diverge to represent groups on one spectrum or the other of the association, the lines would be very close together. Building from these strengths in future research is essential as knowing particularly what experiences are urgently problematic is something that many parents, educators, healthcare providers, judges, and juvenile justice practitioners desperately want to know so that future delinquent behaviors can be prevented. Third, the Add Health study asked respondents *how many times* a respondent experienced maltreatment, rather than a simple “yes” or “no.” Recent evidence indicates the frequency of maltreatment may matter more than the type of maltreatment, as types of maltreatment tend to co-occur [56, 62].

Additionally, while we discussed the weaknesses above of self-report data, it is important to note here that there are also strengths. Specifically, the rates of both maltreatment and offending behavior are higher in Add Health than in government reports. We likely capture experiences here that were not reported. This may indicate that Add Health was successful at giving adolescent’s a sense of confidence and confidentiality in the survey and allowing them to feel safe self-reporting delinquent or criminal behaviors for which they did not get caught. It also may mean that a young person may have shared an experience they felt happened but under further investigation did not justify government reporting. More importantly though, both child welfare investigations and policing are patterned by socioeconomic status and race [12, 57, 58, 63]. This is important because in this study we can capture youth who did not end up in the welfare or justice systems – who are overwhelmingly youth of color [64] – and therefore can create estimates for the associations for a broader range of youth. This strikes us as particularly important given that race is found to be a significant moderator in other administrative data studies [12] but not in some other prospective studies [46] suggesting that more exploration into the potential for bias here is important. We hope that the results here can be compared to studies of administrative data to better inform the field of potential strengths and biases to using both methods of data collection.

Finally, by using linear mixed effects models, we decreased the models’ vulnerability to endogeneity. There are many potential factors that may be shared predictors of both maltreatment and delinquency, and our data source did not allow us to control for all of them. Other studies have used evaluations or natural experiments to find exogenous patterns but linear mixed effects models, by examining an individual’s change over time, controls for those unobserved factors that are time invariant. This robust method allowed us to look at how these associations change when the frequency and types of maltreatment increased, as well as test for differences by sex, race/ethnicity, and sexual orientation.

## Conclusion

Given our interesting findings as well as the strengths and limitations discussed above, further exploration into the relationship between childhood experiences of maltreatment and trauma are needed to better understand critical junctures and potential opportunities to support young people to overcome challenges. Specifically, better understanding the differences in experiences between males and females may be particularly important as it is becoming clearer that more females are becoming engaged in the juvenile justice system and that many males have also experienced maltreatment and trauma. In addition to contributing to literature surrounding these differences, our findings can inform others’ (i.e., police officers, judges, teachers, etc.) views of externalizing behaviors, particularly in males but also for females as they engage with the juvenile justice system more. It is important for all professionals who work with young men – as well as their parents – to recognize that externalizing behavior can be a warning sign of underlying stressors that are impacting a young man’s mental and physical well-being. Teaching adolescent boys and young men to understand and identify their stress responses could decrease their need for externalizing responses that can leave them vulnerable to continued trauma [65]. All youth deserve a system that addresses their hurt and supports them to grow into responsible, healthy adults.

## Supplementary information

**Additional file 1: Table S1.** Regression results from models two, three, and seven for violent offending (model titles described in columns below).

**Additional file 2: Table S2.** Regression results from models two, three, five, seven, and eight for nonviolent offending (model titles described in columns below).

**Additional file 3: Table S3.** Intraclass correlation for both violent and nonviolent offending models comparing baseline and analytic models.

## Data Availability

This research uses data from Add Health, a program project directed by Kathleen Mullan Harris and designed by J. Richard Udry, Peter S. Bearman, and Kathleen Mullan Harris at the University of North Carolina at Chapel Hill, and funded by grant P01-HD31921 from the Eunice Kennedy Shriver National Institute of Child Health and Human Development, with cooperative funding from 23 other federal agencies and foundations. Special acknowledgment is due Ronald R. Rindfuss and Barbara Entwisle for assistance in the original design. Add Health has public-use data files. More information on how to obtain the Add Health data files is available on the Add Health website (http://www.cpc.unc.edu/addhealth). No direct support was received from grant P01-HD31921 for these analyses.

## Abbreviations

Add Health: National Longitudinal Study of Adolescent to Adult Health
ICC: Intraclass correlation coefficient
LGBQ: Lesbian, Gay, Bisexual, or Queer
