# Supplementary material for: Describing associations between child maltreatment frequency and the frequency and timing of subsequent delinquent or criminal behaviors across development: variation by sex, sexual orientation, and race
Source: BMC Public Health. 2019 Nov 12;19:1306. doi: 10.1186/s12889-019-7655-7 (PMC6849276; doi:10.1186/s12889-019-7655-7)
Supplement: Supplementary file 1 — Additional file 1: Table S1. Regression results from models two, three, and seven for violent offending (model titles described in columns below). [file 12889_2019_7655_MOESM1_ESM.docx]

**Additional file 1: Table S1 Regression results from models two, three, and seven for violent offending (model titles described in columns below)**

| Model | Covariates (M2) | | Moderation by Sex (M3) | | Maltreatment as Predictor (M7) |
| --- | --- | --- | --- | --- | --- |
| Maltreatment frequency |  | |  | | 0.16** |
| Age x Maltreatment |  | |  | | -0.01 |
| Age^2 x Maltreatment |  | |  | | <0.01 |
| Age | -0.11*** | | -0.04 | | -0.08*** |
| Age^2 | <0.01*** | | <0.01 | | <0.01** |
| Hispanic (ref: white) | 0.16*** | | 0.17*** | | 0.16*** |
| Black (ref: white) | 0.26*** | | 0.25*** | | 0.25*** |
| Asian (ref: white) | 0.08* | | 0.07* | | 0.06 |
| Native American (ref: white) | 0.19* | | 0.19* | | 0.16 |
| Other race/ethnicity (ref: white) | -0.03 | | -0.02 | | -0.02 |
| Female (ref: male) | -0.38*** | | 0.69* | | -0.38*** |
| Female x Age |  | | -0.13*** | |  |
| Female x Age^2 |  | | <0.01*** | |  |
| LGBQ (ref: heterosexual) | 0.04 | | 0.04 | | 0.02 |
| Public assistance in household before age 18 | 0.07* | | 0.07* | | 0.03 |
| Ever repeated or been held back a grade | 0.22*** | | 0.22*** | | 0.21*** |
| Ever suspended, expelled, or dropped out | 0.20* | | 0.20* | | 0.19* |
| Ever used alcohol, cigarettes, or illicit substances | 0.34*** | | 0.34*** | | 0.32*** |
| Ever in a foster home | 0.15* | | 0.15* | | 0.10 |
| Intercept | 1.83*** | | 1.28*** | | 1.42*** |
| * p<0.05, ** p<0.01, *** p<0.001 | |  | |  |  |
| Note: "<0.001" is used for values (positive or negative) that round to 0.00 | |  | |  |  |
