# Supplementary material for: Describing associations between child maltreatment frequency and the frequency and timing of subsequent delinquent or criminal behaviors across development: variation by sex, sexual orientation, and race
Source: BMC Public Health. 2019 Nov 12;19:1306. doi: 10.1186/s12889-019-7655-7 (PMC6849276; doi:10.1186/s12889-019-7655-7)
Supplement: Supplementary file 2 — Additional file 2: Table S2. Regression results from models two, three, five, seven, and eight for nonviolent offending (model titles described in columns below). [file 12889_2019_7655_MOESM2_ESM.docx]

**Additional file 2: Table S2 Regression results from models two, three, five, seven, and eight for nonviolent offending (model titles described in columns below)**

| Model | Covariates (M2) | Moderation by Sex (M3) | Moderation by Sexual Orientation (M5) | Maltreatment as Predictor (M7) | Moderation by Sex of Maltreatment Predictor (M8) |
| --- | --- | --- | --- | --- | --- |
| Maltreatment Frequency |  |  |  | -1.64*** | -2.77*** |
| Maltreatment x Age |  |  |  | 0.22*** | 0.36*** |
| Maltreatment x Age^2 |  |  |  | -0.01*** | -0.01*** |
| Age | -0.02 | 0.06* | -0.03* | 0.01 | 0.08** |
| Age^2 | <0.01** | <0.01*** | <0.01 | <0.01*** | <0.01*** |
| Hispanic (ref: white) | 0.03 | 0.03 | 0.03 | 0.02 | 0.02 |
| Black (ref: white) | -0.01 | -0.01 | -0.01 | -0.01 | -0.01 |
| Asian (ref: white) | 0.03 | 0.03 | 0.03 | <0.01 | <0.01 |
| Native American (ref: white) | 0.03 | 0.03 | 0.03 | 0.03 | 0.04 |
| Other race/ethnicity (ref: white) | -0.07 | -0.06 | -0.07 | -0.07 | -0.08 |
| Female | -0.46*** | 0.73* | -0.46*** | -0.46*** | 0.65* |
| Female x Age |  | -0.15*** |  |  | -0.13*** |
| Female x Age^2 |  | <0.01*** |  |  | <0.01*** |
| Female x Maltreatment |  |  |  |  | 1.87* |
| Female x Maltreatment x Age |  |  |  |  | -0.23* |
| Female x Maltreatment x Age^2 |  |  |  |  | 0.01* |
| LGBQ | 0.30*** | 0.30*** | -0.96* | 0.28*** | 0.28*** |
| LGBQ x Age |  |  | 0.13** |  |  |
| LGBQ x Age^2 |  |  | <0.01** |  |  |
| Public assistance in household before age 18 | 0.08* | 0.08* | 0.08* | 0.06 | 0.06 |
| Ever repeated or been held back a grade | -0.02 | -0.02 | -0.02 | -0.04 | -0.04 |
| Ever suspended, expelled, or dropped out | 0.23* | 0.23* | 0.23* | 0.21* | 0.21* |
| Ever used alcohol, cigarettes, or illicit substances | 0.46*** | 0.46*** | 0.46*** | 0.43*** | 0.43*** |
| Ever lived in a foster home | 0.13 | 0.13 | 0.13 | 0.11 | 0.11 |
| Intercept | 1.20*** | 0.58* | 1.36*** | 0.82*** | 0.24 |
| * p<0.05, ** p<0.01, *** p<0.001 | | | | | |
| Note: "<0.001" is used for values (positive or negative) that round to 0.00 | | | | | |
