# Supplementary material for: Describing associations between child maltreatment frequency and the frequency and timing of subsequent delinquent or criminal behaviors across development: variation by sex, sexual orientation, and race
Source: BMC Public Health. 2019 Nov 12;19:1306. doi: 10.1186/s12889-019-7655-7 (PMC6849276; doi:10.1186/s12889-019-7655-7)
Supplement: Supplementary file 3 — Additional file 3: Table S3. Intraclass correlation for both violent and nonviolent offending models comparing baseline and analytic models. [file 12889_2019_7655_MOESM3_ESM.docx]

**Additional file 3: Table S3 Intraclass correlation for both violent and nonviolent offending models comparing baseline and analytic models.**

| Violent offending |  | Nonviolent offending |  |
| --- | --- | --- | --- |
| Baseline model (M1) | Maltreatment predictor (M7) | Baseline model (M1) | Maltreatment predictor, moderation by Sex (M8) |
| ICC = 0.16 | ICC = 0.10 | ICC = 0.19 | ICC = 0.13 |
